# Diagnostic and prognostic value of the injury–inflammation–fibrosis serum biomarker panel (KL-6, SAA, YKL-40) in lung cancer treatment-associated ILD

**DOI:** 10.1186/s12890-026-04479-6

**Published:** 2026-07-16

**Authors:** Shuilian Wu, Jing Jin, Lingli Wu, Xiangjiang Zeng, Qiang Hou, Lei Shu

**Affiliations:** Department of Clinical Laboratory, Hangzhou Cancer Hospital, Hangzhou, 310002 China

**Keywords:** Lung cancer, ILD, KL-6, SAA, YKL-40

## Abstract

**Objective:**

Cancer therapy–related interstitial lung disease (CT‑ILD) is a serious and potentially fatal complication in lung cancer patients treated with radiotherapy or antineoplastic agents, characterized by high mortality and limited treatment options. Early recognition remains challenging due to the absence of sensitive and specific biomarkers. This study aimed to establish a tripartite serum biomarker panel—KL‑6 (lung injury), SAA (inflammation), and YKL‑40 (fibrosis)—representing the “injury–inflammation–fibroproliferation” axis, and to evaluate its diagnostic and prognostic value for CT‑ILD.

**Methods:**

In this single-center observational case-control study, we consecutively enrolled hospitalized patients with histologically confirmed lung cancer who underwent thoracic radiotherapy and/or systemic anti-neoplastic therapy between January 2024 and June 2025. Based on imaging findings, patients were classified into the ILD group (*n* = 73) or N‑ILD group (*n* = 67). Serum KL‑6, SAA, and YKL‑40 were quantified alongside conventional inflammatory markers (CRP, IL‑6). ROC analysis evaluated diagnostic performance. Logistic regression identified independent ILD correlates. Subgroup analyses delineated etiological differences, comparing radiotherapy-induced ILD (RT‑ILD) with drug-induced ILD (DI‑ILD), and further explored their associations with clinical benefit as an exploratory endpoint.

**Results:**

KL‑6, SAA, YKL‑40, and IL‑6 were significantly elevated in ILD compared with N‑ILD (all *P* < 0.05). ROC analysis yielded AUCs of 0.764 for KL‑6, 0.880 for SAA, and 0.718 for YKL‑40, with optimal cut‑offs of 349.5 U/L, 18.7 mg/L, and 71.65 ng/mL, respectively (all *P* < 0.001). Independent correlates of ILD included SAA > 18.7 mg/L (OR = 32.321), KL‑6 > 349.5 U/L (OR = 5.842), and YKL‑40 > 71.65 ng/mL (OR = 5.250). In the overall cohort, the combined model (KL-6 + SAA + YKL-40) showed good calibration (Hosmer-Lemeshow *P* > 0.05) but did not significantly outperform SAA alone in AUC (DeLong test, *P* > 0.05). SAA maintained high diagnostic value in both RT-ILD (AUC = 0.834) and DI-ILD (AUC = 0.891) subgroups. In exploratory analyses, higher KL-6 levels and the presence of multiple metastases were associated with a lack of durable clinical benefit (both *P* < 0.05).

**Conclusion:**

The KL‑6/SAA/YKL‑40 biomarker panel demonstrates exploratory utility for early CT‑ILD detection. SAA alone shows high diagnostic performance, while the combined model offers acceptable calibration. The panel may support risk stratification and timely individualized management during chemo‑/radiotherapy.

**Supplementary Information:**

The online version contains supplementary material available at https://doi.org/10.1186/s12890-026-04479-6.

## Introduction

Interstitial lung diseases (ILDs) comprise a heterogeneous group of non-malignant, non-infectious pulmonary disorders characterized predominantly by alveolar inflammation and interstitial fibrosis. Pathologically, some ILDs are driven mainly by inflammatory processes, whereas others manifest as extensive fibrosis, distortion of lung architecture, and reduced lung volumes [1]. According to the Global Burden of Disease data, the worldwide incidence of ILD has increased by 51% over the past decade—from 207.2 cases per 100,000 individuals in 1990 to 313.2 cases in 2019 [2].

In patients with lung cancer, therapy-related ILD may arise from thoracic radiotherapy or systemic antineoplastic agents [3]. These events substantially impair treatment continuity and survival, particularly when acute exacerbations (AE-ILD) occur, which can rapidly lead to respiratory failure and death [4]. Survival in lung cancer patients with ILD is markedly lower than in those without ILD, necessitating heightened safety considerations during treatment. The risk of treatment-induced AE-ILD or pneumonia can range from 5% to 20% [5]. Cancer Therapy-related Interstitial Lung Disease (CT-ILD) typically results from treatment-associated lung injury, encompassing radiation exposure, cytotoxic chemotherapy, and immune checkpoint inhibitor administration [6]. Drug‑induced interstitial lung disease (DI-ILD) is a serious adverse reaction in lung cancer therapy, with the potential to precipitate respiratory failure and mortality [7]. A study reported an ILD incidence of 15.4% among trastuzumab-treated patients [8]. Common antineoplastic agents linked to DI-ILD—such as gemcitabine, docetaxel, methotrexate, and nivolumab—are frequently used as first- or second-line regimens in lung cancer management [6]. A meta-analysis of eight trials involving 644 patients demonstrated an AE-ILD incidence of 19% in chemotherapy arms [9].

Radiotherapy‑induced interstitial lung disease (RT-ILD) represents another major form of CT-ILD, characterized by radiation-associated pulmonary damage. Typical clinical manifestations include dyspnea and dry cough; however, early disease stages often lack specific features, leading to misdiagnosis as infection or pulmonary edema [10]. Current diagnostic approaches rely mainly on high-resolution computed tomography (HRCT), but the insidious nature and subtle radiologic patterns of CT-ILD pose significant challenges to timely detection in lung cancer patients. Thus, achieving early diagnosis of ILD in this context is of critical importance.

Blood-based biomarkers, including tumor-derived molecules and products of metabolic or inflammatory processes, are gaining prominence in translational research. Owing to their ease of acquisition, minimal invasiveness, and strong disease relevance, they are widely accepted in clinical practice. Building on this rationale, the present study aimed to develop a multivariable predictive model for therapy-related ILD in lung cancer patients, anchored in three mechanistic domains: primary lung injury (Krebs von den Lungen-6, KL-6), inflammation (serum amyloid A, SAA), and fibrosis (Chitinase-3-like protein 1, YKL-40). We sought to identify serum biomarkers capable of facilitating early differential diagnosis and prognosis assessment in CT-ILD.

## Materials and methods

### Patients and study design

This was a single‑center prospective observational study with a case–control comparison, conducted at Hangzhou Cancer Hospital between January 2024 and June 2025. Inclusion criteria were: (1) histologically confirmed lung cancer; (2) hospitalized patients receiving thoracic radiotherapy and/or systemic anti‑cancer therapy; (3) baseline high‑resolution computed tomography (HRCT) performed prior to initiation of the index cancer therapy. Exclusion criteria included: (1) pre‑existing ILD findings on baseline HRCT; (2) evidence of acute heart failure (e.g., elevated BNP with cardiomegaly/pulmonary edema on imaging); (3) active pulmonary infection (e.g., positive microbiological tests and/or rapid clinical improvement with antibiotics).

Consecutive eligible patients were enrolled. During hospitalization and treatment monitoring, HRCT was performed when clinically indicated and/or per institutional practice to evaluate new respiratory symptoms and to screen for treatment‑related pulmonary toxicities. The “index HRCT assessment” was defined as the first HRCT demonstrating new ILD for cases, or the post‑treatment HRCT confirming absence of ILD for controls.

ILD diagnosis was adjudicated by a multidisciplinary team (MDT) consisting of pulmonologists and radiologists based on a compatible temporal relationship to cancer therapy, HRCT patterns, clinical presentation, and exclusion of alternative diagnoses [11]. All HRCT images were independently reviewed by two board‑certified thoracic radiologists; discrepancies were resolved by consensus with a third senior thoracic radiologist. Because ILD is a diagnosis of exclusion, we systematically evaluated competing causes. Patients meeting any exclusion criterion (acute heart failure or active pulmonary infection) were not classified as ILD.

For an exploratory comparison, a total of 50 subjects were enrolled into a Normal control (NC) group from the health examination center during the same period. NC participants had no history of malignancy, no respiratory symptoms, and no radiologic evidence of ILD on chest imaging. Individuals with acute infection, autoimmune diseases, or other major inflammatory conditions were excluded. Serum sampling and biomarker measurements followed the same laboratory procedures as in the patient cohort.

### Study endpoints

The primary endpoint was CT‑ILD at the index HRCT assessment during the index hospitalization/treatment monitoring period.

Secondary endpoints included in-hospital mortality.

Durable clinical benefit (DCB) at 6 months was analyzed as an exploratory endpoint and was defined as complete response, partial response, or stable disease lasting at least 6 months according to RECIST version 1.1.

### Definition of ILD subtypes (RT‑ILD vs. DI‑ILD)

For etiological subgroup analyses, ILD cases were further categorized as radiotherapy-related ILD (RT‑ILD) [12] or drug-induced ILD (DI‑ILD) [13] according to the predominant suspected causative exposure, based on the temporal relationship, radiologic distribution (e.g., in-field predominance after thoracic radiotherapy), clinical course, and MDT consensus.

### Sample collection

For ILD cases, peripheral venous blood samples were collected within 48 h after the first radiographic confirmation of ILD on the index HRCT. Whenever feasible, sampling was performed prior to initiation or escalation of systemic corticosteroid therapy. For non‑ILD control subjects, serum samples were collected from patients receiving concurrent inpatient cancer therapy after post‑treatment index HRCT assessment verified the absence of ILD. For each patient, 1.5 mL of peripheral serum from routine clinical sampling was obtained for biomarker assays. Comprehensive clinical data, including demographic characteristics, treatment regimens, and relevant laboratory results, were recorded for all participants.

### Laboratory measurements

Serum levels of Krebs von den Lungen-6 (KL-6), serum amyloid A (SAA), high-sensitivity C-reactive protein (hs-CRP), and chitinase-3-like protein 1 (YKL-40) were measured using a latex-enhanced immunoturbidimetric assay on the Abbott C16000 automated biochemical analyzer (Abbott Diagnostics, USA). Interleukin-6 (IL-6) concentrations were determined via chemiluminescence magnetic microparticle immunoassay using the Meikang MS-i2280 automated chemiluminescence analyzer (Meikang Biotechnology, China). All assays were performed strictly in accordance with the manufacturer’s instructions for instruments and reagent kits, under standard laboratory quality control procedures.

### Statistical analysis

All statistical analyses were performed using IBM SPSS Statistics, Version 26.0 (IBM Corp, Armonk, NY, USA) and R software (version 4.5.1). Continuous data were assessed for normality: normally distributed data were presented as mean ± SD and were compared using Student’s t‑test; non‑normally distributed data are presented as median (interquartile range) and were compared using the Mann‑Whitney U test. Categorical data were compared using the chi‑square test. Correlation analyses used Pearson or Spearman coefficients as appropriate.

Receiver operating characteristic (ROC) curve analysis was utilized to determine the optimal cut-off values, and the DeLong test was applied to compare areas under the curve (AUCs). Both univariate and multivariate logistic regression analyses were performed to identify factors independently associated with ILD at the index assessment. Because pre-treatment baseline serum samples were not available for ILD cases, our regression models were intended to evaluate diagnostic associations at the index time window rather than baseline risk prediction.

Calibration curves for the combined biomarker model were plotted using the rms package in R, with 1000 bootstrap resamples to evaluate agreement between predicted probabilities and observed outcomes. The Hosmer‑Lemeshow goodness‑of‑fit test was also performed. A two‑tailed *P* < 0.05 was considered statistically significant.

## Results

### Baseline characteristics

In this study, the ILD group (interstitial lung disease) and N-ILD group (non‑ILD) were compared. Additionally, patients with DCB and those without (non‑DCB) were analyzed for exploratory outcomes. Baseline clinical characteristics of the enrolled lung cancer patients are summarized in Table 1. A total of 140 hospitalized lung cancer patients were enrolled and stratified into ILD and N-ILD groups according to the occurrence of ILD during hospitalization. Additionally, patients who achieved a DCB following treatment were categorized as the DCB group, whereas those with disease progression were assigned to the non-DCB group. Most patients presented with stage IV lung cancer. Among the entire cohort, 21 had comorbid chronic obstructive pulmonary disease (COPD), and 15 died during hospitalization. Specifically, the ILD subgroup showed a 46.6% disease progression rate, 16 COPD cases, and 14 deaths, compared to 11.9% progression, 5 COPD cases, and 1 death in the N-ILD subgroup. No statistically significant differences were observed in age, gender, pathological type, or performance status (PS) between the two groups.

**Table 1:** Baseline characteristics of the lung cancer patients

	N-ILD(*n* = 67)	ILD(*n* = 73)	Z/χ²	*P* Value
Age	67(60–73)	69(65–73)	-1.608	0.108
Gender			0.888	0.346
(male)	56(83.6%)	65(89.0%)		
(female)	11(16.4%)	8(11.0%)		
Histology			2.324	0.313
SCLC	8(11.9%)	15(20.5%)		
NSCLC				
(SCC)	31(46.3%)	34(46.6%)		
(ADC)	28(41.8%)	24(32.9%)		
TNM stage			0.016	0.899
(I-III)	19(28.4%)	20(27.4%)		
(IV)	48(71.6%)	53(72.6%)		
Multiple metastases	40(59.7%)	50(68.5%)	1.176	0.824
ECOG PS score			/	0.416
(0–2)	66(98.5%)	69(94.5%)		
(3–4)	1(1.5%)	4(5.5%)		
Smoking history, Yes	21(31.3%)	34(46.6%)	3.398	0.065
Radiotherapy	14(20.9%)	21(28.8%)	1.155	0.283
Systemic Therapy	51(76.1%)	55(75.3%)	0.011	0.915
COPD	5(7.5%)	16(21.9%)	5.725	0.017*
DCB			19.956	< 0.001**
(Yes)	59(88.1%)	39(53.4%)		
(No)	8(11.9%)	34(46.6%)		
Death	1(1.5%)	14(19.2%)	9.649	0.001**

Values are numbers (%) or medians (interquartile ranges)

Overlap between the two subgroups was present but not further analyzed due to small sample size

*SCLC* small cell lung cancer, *NSCLC* non-small cell lung cancer, *SCC* squamous cell carcinoma, *ADC* adenocarcinoma, *TNM stage* Tumor-Node-Metastasis stage, *ECOG PS score* Eastern Cooperative Oncology Group performance status score, *COPD* chronic obstructive pulmonary disease, *DCB* durable clinical benefit

* *P* < 0.05 ** *P* < 0.01. some patients received both radiotherapy and chemotherapy; therefore, the sum of RT-ILD and DI-ILD cases exceeds the total ILD number

### Serum levels of KL-6, SAA, and YKL-40

**Fig. 1:**
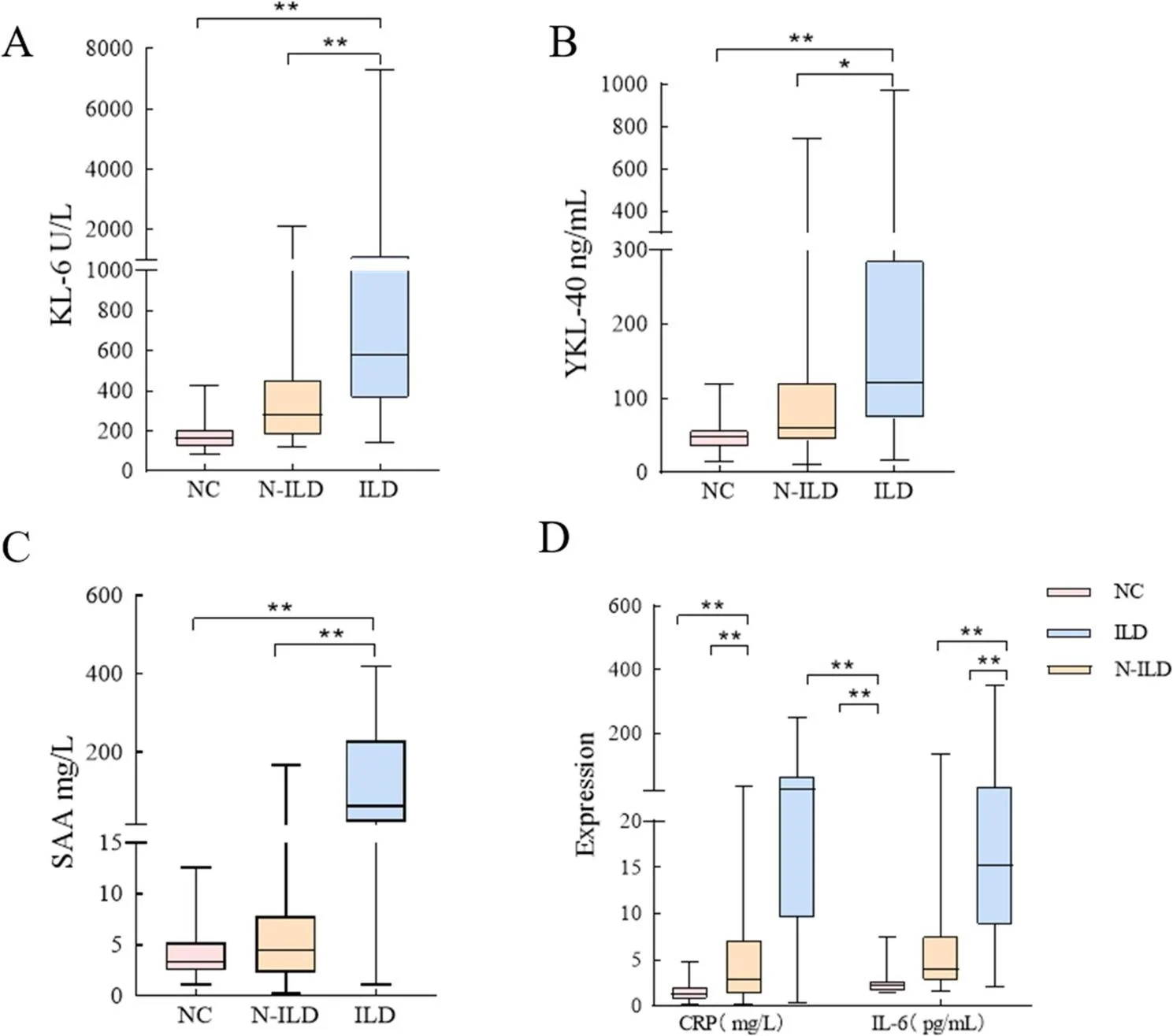
Serum levels of biomarkers across study groups. **A** KL-6 levels; **B** YKL-40 levels; **C** SAA levels; **D** CRP and IL-6 levels

Analysis of serum biomarkers (Fig. 1A and B) revealed that median KL-6 levels were 577.0 U/mL, 279.0 U/mL, and 166.0 U/mL in the ILD, N-ILD, and NC groups, respectively. Corresponding median YKL-40 levels were 121.6 ng/mL, 59.0 ng/mL, and 47.4 ng/mL. The overall distributions of both KL-6 and YKL-40 differed significantly across the three groups (*P* < 0.05). Further measurements of inflammatory markers SAA, CRP, and IL-6 (Fig. 1C and D) also showed statistically significant intergroup differences (*P* < 0.05). Specifically, median SAA concentrations were 63.77 mg/L in the ILD group, 4.50 mg/L in the N-ILD group, and 3.35 mg/L in the NC group.

### Correlation of KL-6 with YKL-40 and inflammatory factors

**Fig. 2:**
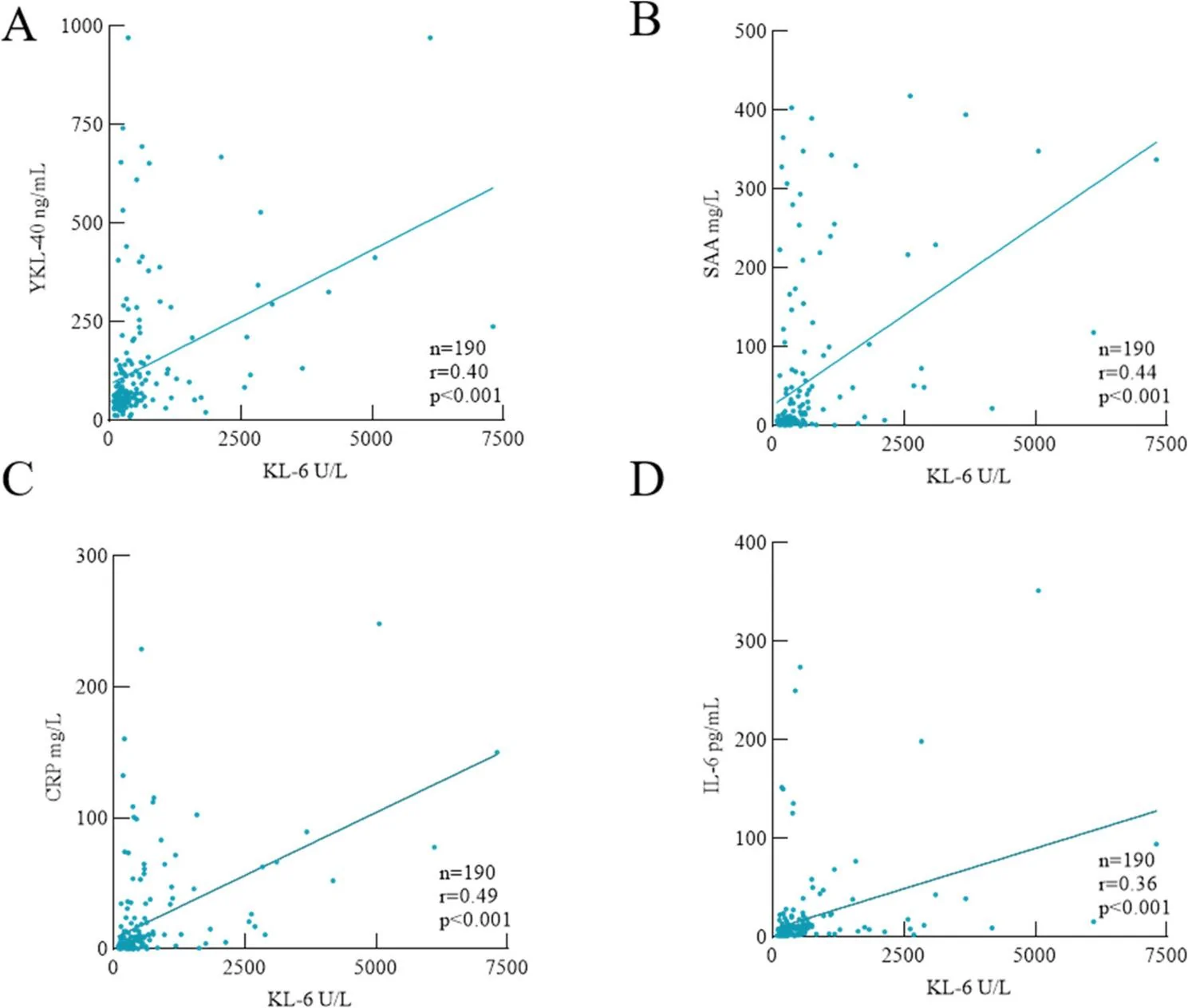
Correlation analysis between KL-6 and other biomarkers. **A** YKL-40 (*r* = 0.40, *P* < 0.001); **B** SAA (*r* = 0.44, *P* < 0.001); **C** CRP (*r* = 0.49, *P* < 0.001); **D** IL-6 (*r* = 0.36, *P* < 0.001)

We assessed the correlations between serum KL-6 and YKL-40, as well as various inflammatory factors, across the ILD, N-ILD, and NC groups. KL-6 levels showed significantly positive correlations with YKL-40 (*r* = 0.40, *P* < 0.001; Fig. 2A), SAA (*r* = 0.44, *P* < 0.001; Fig. 2B), CRP (*r* = 0.49, *P* < 0.001; Fig. 2C), and IL-6 (*r* = 0.36, *P* < 0.001; Fig. 2D).

### Diagnostic performance for ILD in the total lung cancer cohort

**Fig. 3:**
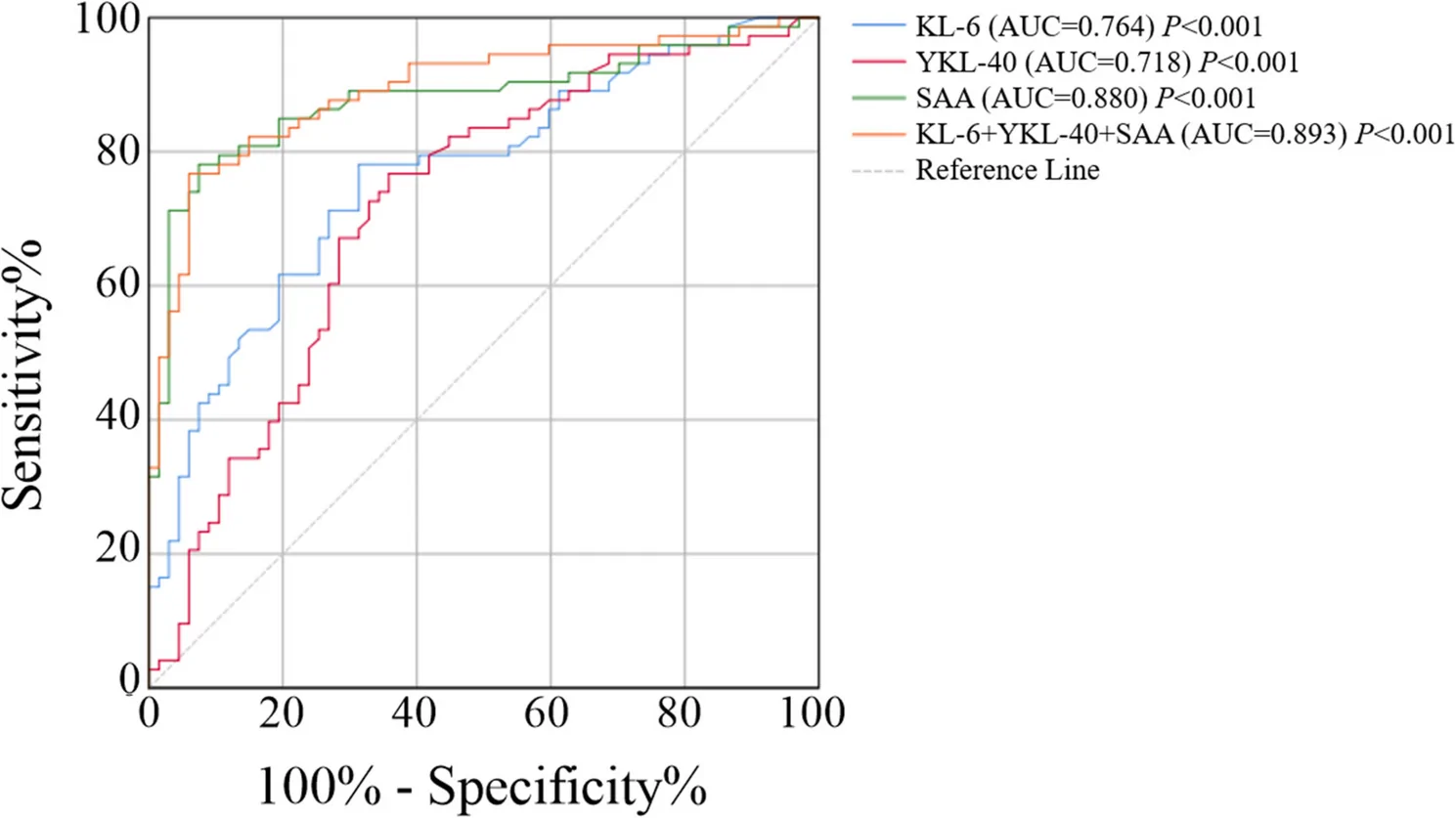
ROC curves of KL-6, YKL-40, SAA, and the combined biomarker panel for discriminating ILD

ROC curve analysis (Table 2; Fig. 3) was performed to evaluate the predictive performance of peripheral blood biomarkers for ILD, using the N-ILD group as controls. The areas under the curve (AUC) for KL-6, YKL-40, and SAA were 0.764, 0.718, and 0.880, respectively. At the maximum Youden index, the optimal cut-off values for KL-6, YKL-40, and SAA were 349.5 U/L, 71.65 ng/mL, and 18.7 mg/L, respectively. KL-6 and SAA demonstrated high diagnostic performance (AUC > 0.75, *P* < 0.001), with SAA being the most notable (AUC = 0.880). The combined prediction of the three biomarkers yielded an AUC of 0.893, with a sensitivity of 76.7% and specificity of 94.0%. Although the combination slightly improved the overall predictive ability, it did not show a significant advantage over SAA alone (Tab. S1).

**Table 2:** Diagnostic performance of serum biomarkers in predicting ILD by ROC analysis

Makers	Cut-off	Sn(%)	Sp(%)	Youden index	AUC	95%CI	*P* Value
KL-6 U/L	349.5	78.1	68.6	0.467	0.764	0.686–0.843	< 0.001**
YKL-40 ng/mL	71.65	76.7	64.1	0.408	0.718	0.632–0.804	< 0.001**
SAA mg/L	18.7	78.1	92.5	0.706	0.880	0.820–0.940	< 0.001**
KL-6 + YKL-40 + SAA	/	76.7	94.0	0.707	0.893	0.839–0.948	< 0.001**

*KL-6* Krebs von den Lungen-6, *YKL-40* Chitinase-3-like protein 1, *SAA* serum amyloid A, *Sn* sensitivity, *Sp* specificity, *AUC* area under the curve, *95%CI* 95% confidence interval

* *P* < 0.05 ** *P* < 0.01

### Independent factors associated with ILD occurrence

**Fig. 4:**
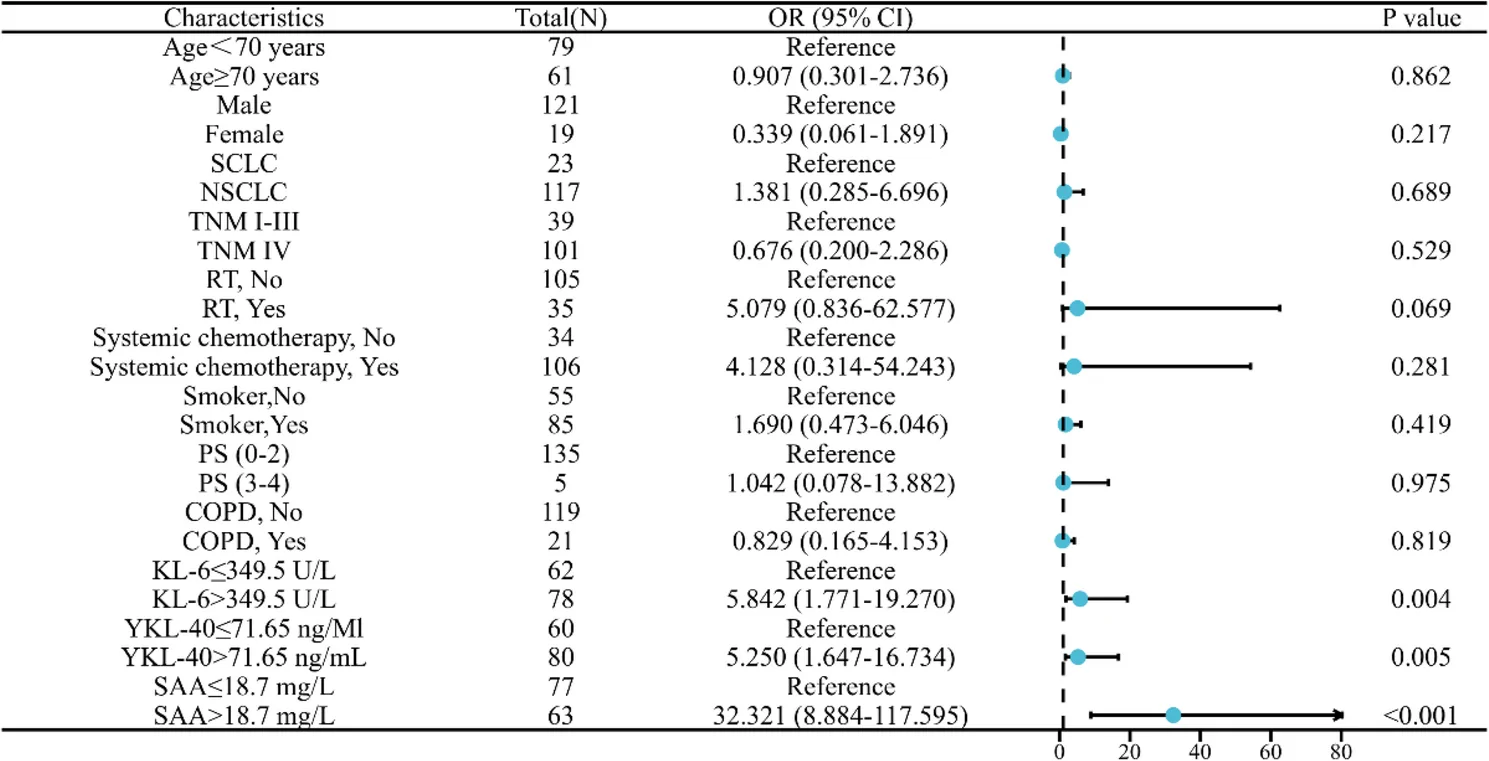
Forest plot illustrating odds ratios (95%CI) for factors independently associated with ILD based on optimal cutoff values determined by ROC analysis

Using the optimal serum cut-off values determined by ROC analysis (KL-6, YKL-40, SAA), we performed multivariate analysis to identify independent correlates of CT‑ILD in lung cancer patients (Fig. 4). As shown in Fig. 4, after adjusting for COPD, KL-6 > 349.5 U/L (odds ratio [OR]: 5.842; 95% confidence interval [CI]: 1.771–19.270; *P* = 0.004), YKL-40 > 71.65 ng/mL (OR: 5.250; 95% CI: 1.647–16.734; *P* = 0.005), and SAA > 18.7 mg/L (OR: 32.321; 95% CI: 8.884-117.595; *P* < 0.001) were all significant independent correlates of ILD, indicating that the predictive value of these biomarkers is independent of COPD. Due to the small number of COPD patients (*n* = 21), further subgroup analyses within COPD status were not performed.

### Subgroup analysis: differentiation between RT-ILD and DI-ILD

**Fig. 5:**
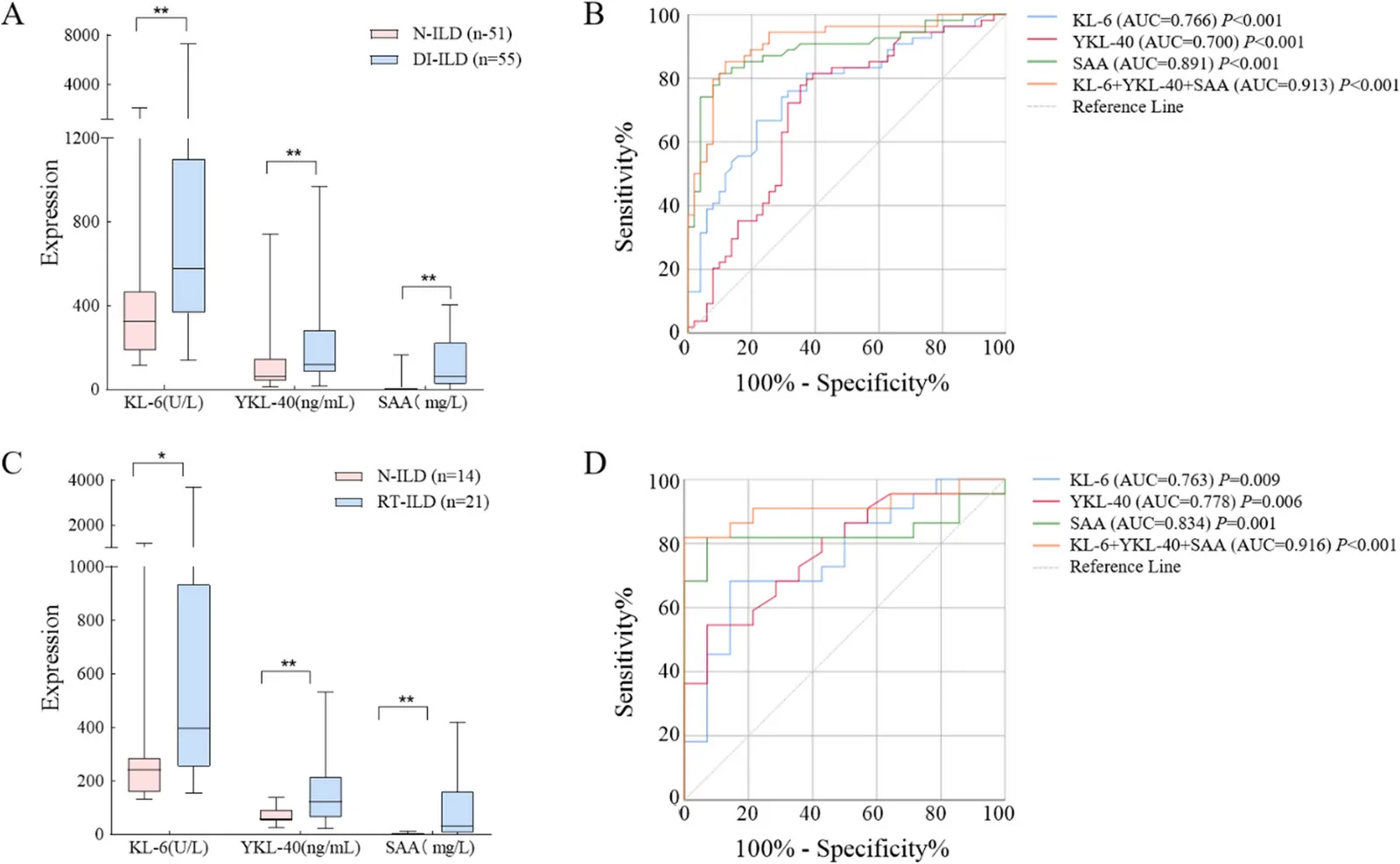
Biomarker levels and diagnostic performance in treatment-specific ILD subgroups. **A** KL-6 and YKL-40 levels in DI-ILD patients. **B** ROC curves of KL-6, YKL-40, SAA, and combined model for distinguishing DI-ILD in the subgroup. **C** KL-6 and YKL-40 levels in RT-ILD patients. **D** ROC curves of KL-6, YKL-40, SAA, and combined model for distinguishing RT-ILD in the subgroup. *P < 0.05, **P < 0.01

Given the potential etiological differences in ILD development among lung cancer patients—including radiation therapy (RT) and drug-induced (DI)—we further evaluated the ability of key serum biomarkers to discriminate between RT-ILD and DI-ILD in the ILD subgroup (Fig. 5A and D; Tables 3 and 4).Among the 140 enrolled patients, 35 received radiotherapy, of whom 21 developed RT-ILD; 106 received systemic chemotherapy, of whom 55 developed DI-ILD (Table 1). In the chemotherapy group, DI-ILD patients showed significantly elevated serum levels of KL-6 (577.0 U/L vs. 327.0 U/L), YKL-40 (121.0 ng/mL vs. 60.00 ng/mL), and SAA (63.77 mg/L vs. 4.80 mg/L) compared to N-ILD patients (all *p* < 0.05; Fig. 5A). Similarly, in the radiotherapy group, RT-ILD patients exhibited significantly higher levels of KL-6 (398.0 U/L vs. 242.0 U/L), YKL-40 (121.6 ng/mL vs. 59.50 ng/mL), and SAA (39.10 mg/L vs. 3.70 mg/L) than N-ILD patients (all *p* < 0.05; Fig. 5C).Notably, no statistically significant differences were observed in serum levels of KL-6 (398.0 U/L vs. 577.0 U/L, *P* = 0.305), YKL-40 (121.6 ng/mL vs. 121.0 ng/mL, *P* = 0.551), or SAA (39.10 mg/L vs. 63.77 mg/L, *P* = 0.283) between the RT-ILD and DI-ILD subgroups (Table 5).

**Table 3:** Predictive value of serum KL-6, YKL-40, and SAA for DI-ILD

Makers	Cut-off	Sn(%)	Sp(%)	Youden index	AUC	95%CI	*P* Value
KL-6 (U/L)	486.0	66.7	78.4	0.451	0.766	0.675–0.857	< 0.001**
YKL-40 (ng/mL)	79.00	77.8	64.7	0.425	0.700	0.597–0.802	< 0.001**
SAA (mg/L)	19.84	81.5	90.2	0.717	0.891	0.826–0.956	< 0.001**
KL-6 + YKL-40 + SAA	/	85.2	88.2	0.734	0.913	0.856–0.969	< 0.001**

*KL-6* Krebs von den Lungen-6, *YKL-40* Chitinase-3-like protein 1, *SAA* serum amyloid A, *Sn* sensitivity, *Sp* specificity, *AUC* area under the curve, *95%CI* 95% confidence interval

* *P* < 0.05 ** *P* < 0.01

**Table 4:** Predictive value of serum KL-6, YKL-40, and SAA for RT-ILD

Makers	Cut-off	Sn(%)	Sp(%)	Youden index	AUC	95%CI	*P* Value
KL-6 U/L	335.0	68.2	85.7	0.539	0.763	0.603–0.923	0.009**
YKL-40 ng/mL	121.3	54.5	92.9	0.474	0.778	0.626–0.929	0.006**
SAA mg/L	6.55	81.8	92.9	0.747	0.834	0.692–0.977	0.001**
KL-6 + YKL-40 + SAA	/	81.8	100	0.818	0.916	0.818-1.000	< 0.001**

*KL-6* Krebs von den Lungen-6, *YKL-40* Chitinase-3-like protein 1, *SAA* serum amyloid A, *Sn* sensitivity, *Sp* specificity, *AUC* area under the curve, *95%CI* 95% confidence interval

* *P* < 0.05 ** *P* < 0.01

**Table 5:** Comparison of serum KL-6, YKL-40, and SAA levels between DI-ILD and RT-ILD

Makers	RT-ILD	DI-ILD	*P* Value
KL-6 U/L	398.0	577.0	0.305
YKL-40 ng/mL	121.6	121.0	0.551
SAA mg/L	39.10	63.77	0.283

*KL-6* Krebs von den Lungen-6, *YKL-40* Chitinase-3-like protein 1, *SAA* serum amyloid A

In the DI-ILD subgroup, SAA demonstrated the highest discriminatory power (AUC = 0.891, *P *< 0.001), with an optimal cut-off value of 19.84 mg/L corresponding to a sensitivity of 81.5% and specificity of 90.2%. KL-6 (cut-off 486.0 U/L, AUC = 0.766, *P* < 0.001) and YKL-40 (cut-off 79.0 ng/mL, AUC = 0.700, *P* < 0.001) also showed moderate diagnostic performance. Similarly, in the RT-ILD subgroup, SAA again performed best (cut-off 6.55 mg/L, AUC = 0.834,* P* < 0.001) with 81.8% sensitivity and 92.9% specificity. KL-6 (cut-off 335.0 U/L, AUC = 0.763, *P* = 0.009) and YKL-40 (cut-off 121.3 ng/mL, AUC = 0.778, *P* = 0.006) also demonstrated moderate to good discriminatory value. Although the combined three-biomarker panel (KL-6, YKL-40, SAA) achieved AUCs of 0.913 (85.2% sensitivity, 88.2% specificity) for DI-ILD and 0.916 (81.8% sensitivity, 100% specificity) for RT-ILD compared with any single biomarker, DeLong’ s test showed that the differences in AUC between the panel and SAA were not statistically significant. (Tab. S2-S3). Post‑hoc power analysis confirmed adequate statistical power for SAA and the combined model in the overall and subgroup analyses (Tab. S4).

### Serum KL-6, YKL-40, and SAA levels predict clinical outcomes in lung cancer patients

**Fig. 6:**
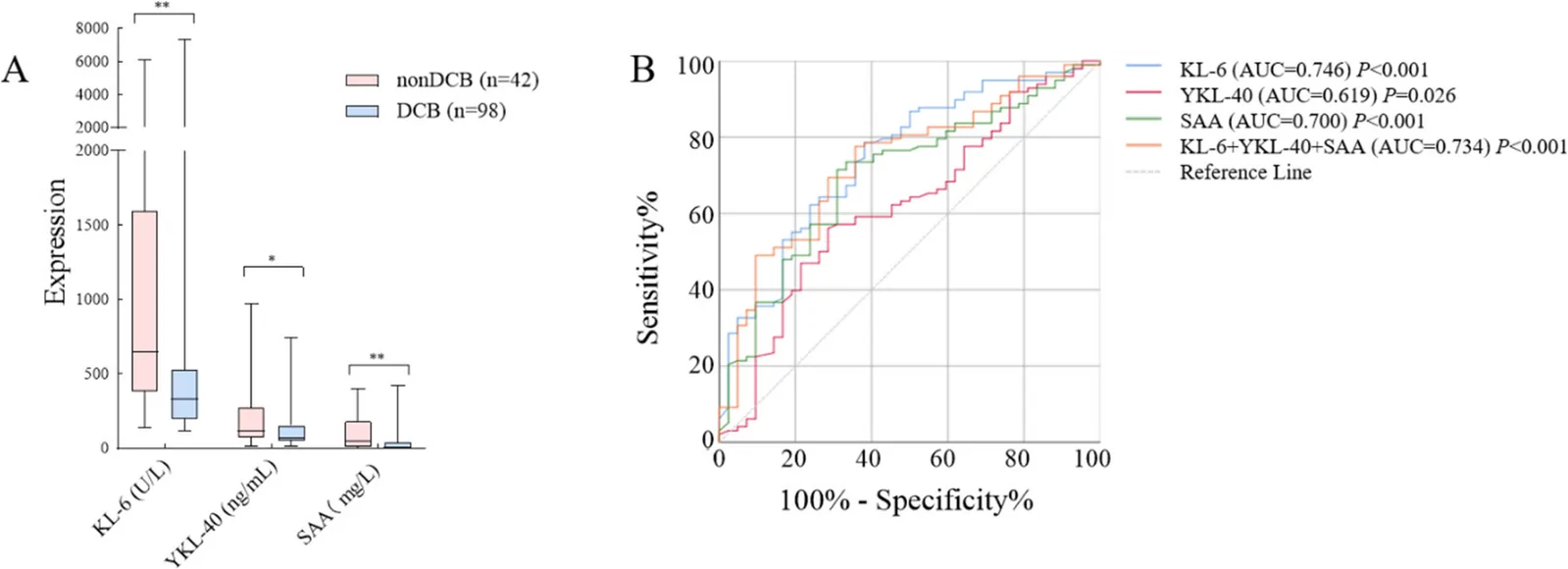
Biomarker levels and performance in predicting durable clinical benefit. **A** Serum KL-6, YKL-40, and SAA levels in DCB versus non-DCB patients; **B** ROC curves showing cut-off levels of serum KL-6, YKL-40, and SAA for predicting DCB

Having established the diagnostic value of serum KL-6, YKL-40, and SAA for ILD and their ability to differentiate between treatment-related subtypes, we next evaluated their utility in association with clinical outcomes—specifically, DCB—in ILD patients. Follow-up data were recorded from the time of initial serum collection during hospitalization. Among the 140 lung cancer patients followed for 6 months, 98 achieved disease remission or stability (DCB group), while 42 experienced disease progression (non-DCB group).Lower serum levels of KL-6 (328.5 U/L vs. 645.0 U/L), YKL-40 (73.0 ng/mL vs. 118.7 ng/mL), and SAA (7.505 mg/L vs. 48.65 mg/L) were significantly associated with DCB (all *P* < 0.05; Fig. 6A). In this exploratory analysis, ROC curve analysis was further performed to assess the predictive performance of these biomarkers for DCB (Fig. 6B; Table 6). KL-6 demonstrated the highest predictive power among individual markers, with an optimal cut-off of 565.0 U/L, AUC = 0.746 (95% CI: 0.658–0.834, *P* < 0.001), sensitivity of 78.6%, and specificity of 61.9%. The three-biomarker panel achieved an AUC of 0.734 (95% CI: 0.646–0.822, *P* < 0.001), which was marginally higher than that of KL-6 alone, though the difference was not statistically significant (Tab. S5). In this cohort, KL-6 alone effectively identified patients who achieved durable clinical benefit, while YKL-40 and SAA provided supplementary prognostic information. In addition, calibration curves for the above four models (overall ILD, DI‑ILD, RT‑ILD, and DCB prediction) demonstrated good calibration (Hosmer‑Lemeshow test all *P* > 0.05; see Supplementary Fig. S1–S4).

**Table 6:** Predictive value of serum KL-6, YKL-40, and SAA for prognosis in lung cancer patients

Makers	Cut-off	Sn(%)	Sp(%)	Youden index	AUC	95%CI	*P* Value
KL-6 U/L	565.0	78.6	61.9	0.405	0.746	0.658–0.834	< 0.001**
YKL-40 ng/mL	85.3	56.1	71.4	0.275	0.619	0.517–0.721	0.026*
SAA mg/L	25.81	71.4	69.0	0.404	0.700	0.607–0.792	< 0.001**
KL-6 + YKL-40 + SAA	/	77.6	64.3	0.419	0.734	0.646–0.822	< 0.001**

*KL-6* Krebs von den Lungen-6, *YKL-40* Chitinase-3-like protein 1, *SAA* serum amyloid A, *Sn* sensitivity, *Sp* specificity, *AUC* area under the curve, *95%CI* 95% confidence interval

* *P* < 0.05 ** *P* < 0.01

The association between COPD and DCB was further examined (Tab. S6). Univariate logistic regression showed no significant association between COPD and DCB (OR = 0.650, 95% CI: 0.247–1.708, *P* = 0.380). In the multivariable model, only Multiple metastases (OR = 0.377, 95% CI: 0.143–0.994, *P* = 0.048) and KL‑6 > 565.0 U/L (OR = 0.337, 95% CI: 0.137–0.828, *P* = 0.018) remained independent correlates of DCB, whereas SAA, YKL‑40, and ILD status were not statistically significant.

## Discussion

CT-ILD represents a serious and often fatal complication arising from radiotherapy and pharmacotherapy in lung cancer patients, with early diagnosis and accurate prognosis remaining major clinical challenges [14]. We selected KL‑6, SAA, and YKL‑40 to represent complementary biological domains along an “injury–inflammation–fibroproliferation” axis relevant to CT‑ILD. KL‑6 (a MUC1 mucin) is mainly derived from injured or regenerating alveolar type II epithelial cells and has been widely used as a circulating biomarker in ILD [15, 16]. Notably, serum KL‑6 can show time-dependent kinetics after acute lung injury, and peak levels may occur days after clinical onset, which underscores the importance of defining the sampling window [17]. SAA is an acute-phase reactant reflecting systemic inflammation and has also been investigated in radiation-associated lung injury contexts [18]. YKL‑40, a chitinase-like protein involved in tissue remodeling, is elevated in fibrotic ILDs such as idiopathic pulmonary fibrosis (IPF) and has been associated with survival, supporting its relevance to fibroproliferative responses [19, 20]. In this study, we comprehensively evaluated the utility of this three‑domain serum biomarker panel – with KL‑6 representing epithelial injury, SAA representing systemic inflammation, and YKL‑40 representing fibroproliferative remodeling – for the early detection, etiological differentiation, and diagnostic support of ILD during hospitalization. Our findings provide new perspectives for individualized management and prevention. SAA demonstrated outstanding performance in predicting ILD, achieving an AUC of 0.880, with 78.08% sensitivity and 92.54% specificity. As a hepatocyte-derived acute-phase protein, SAA levels rise rapidly within 4–6 h after tissue injury, exhibiting a wider dynamic range and superior tissue penetrance compared to CRP [21, 22]. The strong correlation observed between SAA and KL-6 (*r* = 0.44, *P* < 0.001) suggests SAA may be actively involved in the pathogenesis of ILD.

Radiation- or drug-induced lung injury may result from both direct cytotoxic effects and immune-mediated pathways. Alveolar macrophages non-specifically uptake and degrade pharmaceutical agents, releasing toxic payloads that trigger cytokine-mediated lung injury [23]. This process is closely associated with macrophage infiltration and elevated SAA levels. SAA can stimulate M1 macrophage polarization and upregulate pro-inflammatory cytokines such as IL-6 and IL-1β [24, 25]. Emerging evidence proposes SAA as a protein biomarker of radiation exposure. Studies have shown increased serum SAA levels in mice following thoracic irradiation. SAA activates aberrantly infiltrated macrophages in the lungs via the formyl peptide receptor 2 (FPR2), ultimately promoting fibrotic progression in pulmonary epithelial cells [24]. Additionally, post-radiotherapy SAA can stimulate ROS generation, causing bone marrow injury, with intracellular ROS levels escalating in correlation with SAA concentrations [26]. Furthermore, SAA recruits neutrophils into the pulmonary interstitium and activates the NLRP3 inflammasome, thereby amplifying the IL-1β/IL-6 cascade and further disrupting the alveolar epithelial barrier [27]. This process may indirectly lead to the release of KL-6. The underlying mechanism potentially explains the marked elevation of SAA in ILD and its specificity for tissue injury.

In the overall cohort, KL-6, a recognized marker of type II alveolar epithelial cell injury [28], showed a two-fold increase in median level in the ILD group (577.0 U/L) compared to the N-ILD group (279.0 U/L) (*P* < 0.001). This finding aligns with previous reports indicating that KL-6—a marker of type II cell damage and repair—is overexpressed during ILD, with its serum levels rising in parallel due to increased vascular permeability [29, 30]. Meanwhile, KL-6 promotes fibrosis by activating the fibroblast growth factor (FGF) pathway, and serial monitoring of KL-6 can be used to assess ILD progression and prognosis [31]. Concurrently, YKL-40, a macrophage-secreted pro-fibrotic factor [32], was significantly elevated in the ILD group (121.6 ng/mL) relative to controls (59.0 ng/mL) and showed a moderate correlation with KL-6 (*r* = 0.40). YKL-40 primarily reflects extracellular matrix remodeling and immune activation. Under oxidative lung injury, it participates in tissue restructuring and promotes both pulmonary fibrosis and tissue repair [33, 34]. It is also elevated in ILD and plays a role in chronic obstructive pulmonary disease. However, its diagnostic performance (AUC = 0.718) was slightly lower than that of SAA and KL-6, possibly reflecting its role as a later event in the fibrotic process.

In the etiology-specific subgroup analysis, SAA demonstrated the highest discriminatory power for both RT-ILD and DI-ILD, with AUCs of 0.834 and 0.891, respectively. KL-6 and YKL-40 also showed moderate to good diagnostic efficacy. Notably, the three-biomarker panel achieved AUCs exceeding 0.91 in both scenarios, providing complementary value for etiological differentiation. Although no statistically significant differences in biomarker levels were observed between RT-ILD and DI-ILD subgroups—potentially due to sample size limitations, case mix heterogeneity, or varied treatment regimens—the overall trend consistently indicated elevated systemic inflammation and lung injury markers across different treatment-related ILD subtypes. Although the AUC of the combined model (KL‑6 + SAA + YKL‑40) was numerically higher than that of SAA alone, the DeLong test showed no statistically significant difference (*P* > 0.05 for all comparisons). Nevertheless, calibration curves demonstrated good agreement between predicted probabilities and observed outcomes, indicating acceptable calibration of the combined model. Thus, while SAA alone is a highly effective and practical biomarker for ILD detection in this cohort, the combined model may offer complementary value in terms of calibration, particularly in clinical scenarios requiring accurate probability estimation.

In the prognosis-specific subgroup analysis, this study investigated the predictive role of KL-6, YKL-40, and SAA in DCB. KL-6 alone demonstrated the best performance in predicting DCB (AUC = 0.746). Although KL‑6 is known to be elevated as a tumor marker, its association with DCB in the context of ILD remains hypothesis‑generating. Elevated KL-6 levels may indicate more severe or persistent lung injury and fibrotic progression [35, 36], which could subsequently impair patient tolerance to anti-cancer therapies and compromise treatment sustainability [37]. Furthermore, the combined model in our analysis did not significantly outperform KL-6 alone, possibly due to inter-biomarker correlations and the relatively limited independent contribution of certain markers in prognostic assessment. In summary, KL-6 maintains independent and stable clinical value in prognostic evaluation.

This study provides a systematic analysis of biomarker profiles in lung cancer patients with ILD, integrating clinical data for multidimensional assessment covering three core clinical aspects: early detection, etiological differentiation, and prognostic prediction. However, several limitations should be acknowledged. First, fibrosis and inflammation occur along a continuum. Our samples were collected around the time of CT‑ILD diagnosis and therefore primarily reflect early or subacute lung injury and remodeling activity rather than established chronic fibrosis. Longitudinal HRCT and pulmonary function follow‑up, as well as serial biomarker measurements, are needed to validate whether these biomarkers predict long‑term fibrotic sequelae. Second, the single-center prospective observational design may introduce selection bias. Of note, the proportion of COPD was significantly higher in the ILD group than in the N‑ILD group (21.9% vs. 7.5%, *P* = 0.017). COPD itself can lead to airway inflammation, lung epithelial injury, and elevated systemic inflammation, which may confound serum KL‑6, SAA, and YKL‑40 levels as well as clinical outcomes. Although we adjusted for COPD in the multivariable analysis, the small number of COPD patients (*n* = 21) precluded adequate subgroup analyses or propensity score matching. Moreover, the limited sample size (especially RT‑ILD with only 21 cases) might affect statistical robustness, and the absence of longitudinal monitoring precluded analysis of temporal relationships between biomarker dynamics and disease progression. Future studies should validate these findings in multicenter, large-scale prospective cohorts and further explore the feasibility of implementing the multi-biomarker panel in clinical workflows.

In summary, serum KL-6, YKL-40, and SAA demonstrate potential clinical value in risk assessment, etiology discrimination, and prognostic prediction of ILD in lung cancer patients. SAA showed superior individual performance in etiological differentiation, while KL-6 exhibited robust independence in prognostic evaluation. The three-biomarker panel further improved diagnostic accuracy for etiology classification. These findings offer important insights for early identification, precise stratification, and personalized management strategies in high-risk populations.

## Conclusion

The serum biomarkers SAA, KL-6, and YKL-40 collectively form a “injury–inflammation–fibrosis” triad for identifying CT-ILD. Among them, SAA—with its exceptional diagnostic performance (AUC = 0.880) and high specificity (92.54%)—shows promise as a first-line tool for screening high-risk patients. Integrated with the prognostic value of KL-6 and YKL-40, this panel may help establish a comprehensive management pathway from early warning to intervention, ultimately expanding the therapeutic safety window for lung cancer patients. 

## Supplementary Information

Supplementary Material 1.

## Data Availability

The datasets used and/or analysed during the current study are available from the corresponding author on reasonable request.

## Abbreviations

SCLC: Small cell lung cancer
NSCLC: Non-small cell lung cancer
SCC: Squamous cell carcinoma
ADC: Adenocarcinoma
TNM stage: Tumor–Node–Metastasis stage
ECOG PS score: Eastern Cooperative Oncology Group performance status score
ILD: Interstitial lung disease
DI-ILD: Drug‑induced interstitial lung disease
RT-ILD: Radiotherapy‑induced interstitial lung disease
COPD: Chronic obstructive pulmonary disease
DCB: Durable clinical benefit
ROC: Receiver Operating Characteristic curve
Sn: Sensitivity
Sp: Specificity
AUC: Area under the curve
95%CI: 95% confidence interval
