# Supplementary material for: Diagnostic and prognostic value of the injury–inflammation–fibrosis serum biomarker panel (KL-6, SAA, YKL-40) in lung cancer treatment-associated ILD
Source: BMC Pulm Med. 2026 Jul 16;26:464. doi: 10.1186/s12890-026-04479-6 (PMC13660003; doi:10.1186/s12890-026-04479-6)
Supplement: Supplementary file 1 [file 12890_2026_4479_MOESM1_ESM.docx]

**Supplementary Materials**

In all calibration curves, the dashed diagonal line (Ideal) represents perfect calibration; the dotted line (Apparent) is the unadjusted curve; the solid line (Bias‑corrected) is derived from 1000 bootstrap resamples; shaded areas indicate 95% confidence intervals.

**Fig. S1 Calibration curve of the combined model (KL‑6, SAA, YKL‑40) for differentiating ILD from non‑ILD.** The Hosmer‑Lemeshow test indicated good calibration (χ2=1.579; *P*=0.209).

**Fig. S2 Calibration curve of the combined model (KL‑6, SAA, YKL‑40) for differentiating DI‑ILD from non‑DI‑ILD.** The Hosmer‑Lemeshow test indicated good calibration (χ2=1.354; *P*=0.245).

**Fig. S3 Calibration curve of the combined model (KL‑6, SAA, YKL‑40) for differentiating RT‑ILD from non‑RTILD**. The Hosmer‑Lemeshow test indicated good calibration (χ2=1.971; *P*=0.578).

**Fig. S4 Calibration curve of the combined model (KL‑6, SAA, YKL‑40) for differentiating DCB from non‑DCB**. The Hosmer‑Lemeshow test indicated good calibration (χ2=1.462; *P*=0.227).

**Table S1. DeLong test for AUC differences between single biomarkers and the combined model in predicting ILD.**

| Comparison | ΔAUC | Z | 95%CI | *P* Value |
| --- | --- | --- | --- | --- |
| Combined model vs KL-6 | 0.13 | 3.74 | 0.06 - 0.20 | <0.001** |
| Combined model vs YKL-40 | 0.18 | 3.79 | 0.08 - 0.27 | <0.001** |
| Combined model vs SAA | 0.01 | 0.72 | -0.02 - 0.05 | 0.470 |

**Note**: Data are presented as the difference in area under the curve (ΔAUC) between each individual biomarker (KL-6, YKL-40, SAA) and the combined panel (KL-6+SAA+YKL-40). The Z statistic and 95% confidence interval (CI) for the difference were calculated using DeLong’ s method for paired ROC curves. A positive ΔAUC indicates a higher AUC for the combined panel compared to the individual biomarker. *P*-values < 0.05 were considered statistically significant. * *P*<0.05 ** *P*<0.01

**Table S2. DeLong test for AUC differences between single biomarkers and the combined model in predicting DI-ILD.**

| Comparison | ΔAUC | Z | 95%CI | *P* Value |
| --- | --- | --- | --- | --- |
| Combined model vs KL-6 | 0.15 | 3.52 | 0.07 - 0.23 | <0.001** |
| Combined model vs YKL-40 | 0.21 | 3.74 | 0.10 - 0.33 | <0.001** |
| Combined model vs SAA | 0.02 | 1.02 | -0.02 - 0.06 | 0.306 |

**Note**: Data are presented as the difference in area under the curve (ΔAUC) between each individual biomarker (KL-6, YKL-40, SAA) and the combined panel (KL-6+SAA+YKL-40). The Z statistic and 95% confidence interval (CI) for the difference were calculated using DeLong’ s method for paired ROC curves. A positive ΔAUC indicates a higher AUC for the combined panel compared to the individual biomarker. *P*-values < 0.05 were considered statistically significant. * *P*<0.05 ** *P*<0.01

**Table S3. DeLong test for AUC differences between single biomarkers and the combined model in predicting RT-ILD.**

| Comparison | ΔAUC | Z | 95%CI | *P* Value |
| --- | --- | --- | --- | --- |
| Combined model vs KL-6 | 0.15 | 1.77 | -0.02 - 0.32 | 0.073 |
| Combined model vs YKL-40 | 0.14 | 1.83 | -0.01 - 0.27 | 0.065 |
| Combined model vs SAA | 0.08 | 1.46 | -0.03 - 0.19 | 0.140 |

**Note**: Data are presented as the difference in area under the curve (ΔAUC) between each individual biomarker (KL-6, YKL-40, SAA) and the combined panel (KL-6+SAA+YKL-40). The Z statistic and 95% confidence interval (CI) for the difference were calculated using DeLong’ s method for paired ROC curves. A positive ΔAUC indicates a higher AUC for the combined panel compared to the individual biomarker. *P*-values < 0.05 were considered statistically significant. * *P*<0.05 ** *P*<0.01

**Table S4. Post‑hoc power analysis for the primary diagnostic indicators (SAA and the combined model) in the overall and subgroup analyses**

| Analysis | AUC | Cases (n) | Controls (n) | Power |
| --- | --- | --- | --- | --- |
| Primary (overall ILD, SAA) | 0.880 | 73 | 67 | > 99.9% |
| Primary (overall ILD, Combined model) | 0.893 | 73 | 67 | > 99.9% |
| Subgroup (RT‑ILD, SAA) | 0.834 | 21 | 14 | 99.9% |
| Subgroup (RT‑ILD, Combined model) | 0.916 | 21 | 14 | > 99.9% |
| Subgroup (DI‑ILD, SAA) | 0.717 | 55 | 51 | 99.7% |
| Subgroup (DI‑ILD, Combined model) | 0.913 | 55 | 51 | > 99.9% |

**Note**: ILD, interstitial lung disease; RT-ILD, radiotherapy‑induced interstitial lung disease; DI-ILD, drug‑induced interstitial lung disease; SAA, serum amyloid APower calculated using the Hanley‑McNeil method with two‑sided α = 0.05.

**Table S5. DeLong test for AUC differences between single biomarkers and the combined model in predicting prognosis of lung cancer patients.**

| Comparison | ΔAUC | Z | 95%CI | *P* Value |
| --- | --- | --- | --- | --- |
| Combined model vs KL-6 | -0.01 | -0.36 | -0.08 - 0.05 | 0.717 |
| Combined model vs YKL-40 | 0.12 | 2.64 | 0.03 - 0.20 | 0.008** |
| Combined model vs SAA | 0.03 | 0.95 | -0.04 - 0.11 | 0.343 |

**Note**: Data are presented as the difference in area under the curve (ΔAUC) between each individual biomarker (KL-6, YKL-40, SAA) and the combined panel (KL-6+SAA+YKL-40). The Z statistic and 95% confidence interval (CI) for the difference were calculated using DeLong’ s method for paired ROC curves. A positive ΔAUC indicates a higher AUC for the combined panel compared to the individual biomarker. *P*-values < 0.05 were considered statistically significant. * *P*<0.05 ** *P*<0.01

**Table S6. Univariate and multivariable logistic regression analysis for factors associated with DCB**

| Vairable | OR | Univariate Analysis for 95% CI | *P* Value | OR | Multivariate Analysis for 95% CI | *P* Value |
| --- | --- | --- | --- | --- | --- | --- |
| Age | 0.993 | 0.951-1.037 | 0.090 | - | - | - |
| Gender | 0.698 | 0.254-1.919 | 0.485 | - | - | - |
| Histology | 1.025 | 0.388-2.710 | 0.002** | 0.778 | 0.258-2.349 | 0.656 |
| TNM stage | 0.509 | 0.211-1.226 | 0.128 | - | - | - |
| Multiple metastases | 0.314 | 0.132-0.747 | 0.007** | 0.377 | 0.143-0.994 | 0.048* |
| ECOG PS score | 0.271 | 0.044-1.684 | 0.136 | - | - | - |
| Smoking history | 1.955 | 0.897-4.261 | 0.089 | - | - | - |
| Radiotherapy | 1.096 | 0.472-2.546 | 0.831 | - | - | - |
| Systemic Therapy | 1.157 | 0.504-2.658 | 0.731 | - | - | - |
| COPD | 0.650 | 0.247-1.708 | 0.380 | - | - | - |
| ILD | 0.156 | 0.065-0.371 | 0.000** | 0.322 | 0.097-1.067 | 0.064 |
| KL-6 >565.0U/L | 0.168 | 0.076-0.369 | 0.000** | 0.337 | 0.137-0.828 | 0.018* |
| YKL-40 >85.3 ng/mL | 0.313 | 0.143-0.682 | 0.003** | 0.791 | 0.302-2.072 | 0.633 |
| SAA>25.81 mg/L | 0.179 | 0.082-0.394 | 0.000** | 0.634 | 0.205-1.961 | 0.429 |

**Note**: DCB, durable clinical benefit; TNM stage, Tumor-Node-Metastasis stage; ECOG PS score, Eastern Cooperative Oncology Group performance status score; COPD, chronic obstructive pulmonary disease; ILD, interstitial lung disease; KL-6, Krebs von den Lungen-6; YKL-40, Chitinase-3-like protein 1; SAA, serum amyloid A; OR, odds ratio; CI, confidence interval. Only variables with univariate P < 0.05 were entered into the multivariable logistic regression model. * *P*<0.05 ** *P*<0.01
